# Publisher’s Note: Continued Publication of *Nursing Reports* by MDPI

**DOI:** 10.3390/nursrep10010001

**Published:** 2020-07-29

**Authors:** Franck Vazquez

**Affiliations:** MDPI, St. Alban-Anlage 66, CH-4052 Basel, Switzerland; vazquez@mdpi.com

*Nursing Reports* was launched in 2011 and published over the past nine years by PAGEPress Publications [1]. Prof. Dr. Marilynne N. Kirshbaum, who launched this journal in 2011, served as the founding Editor-in-Chief [2]. In 2018, Dr. Colleen E. Marzilli became Editor-in-Chief. We have been very happy working with the new Editor-in-Chief Prof. Dr. Richard Gray in continuing the publication of this journal [3]. 

MDPI was founded in 1996 as a nonprofit project for the promotion and preservation of the diversity of chemical compounds [4]. Since the first journal, *Molecules* [5], was launched, MDPI’s team has focused on serving academic communities’ ever-growing need to quickly and openly publish the results of their research. MDPI’s portfolio has considerably expanded in the last few years and today totals 250 journals in all fields and disciplines [6], including more than 40 in the healthcare area. 

To ensure that we serve all academic communities well, we are delighted to take over *Nursing Reports* from PAGEPress and continue to publish the journal. 

We will publish two quarterly issues in 2020, and regularly publish four quarterly issues in 2021. 

Enjoy publishing in *Nursing Reports*! 
